# Impact of investigational microbiota therapeutic RBX2660 on the gut microbiome and resistome revealed by a placebo-controlled clinical trial

**DOI:** 10.1186/s40168-020-00907-9

**Published:** 2020-08-31

**Authors:** Suryang Kwak, JooHee Choi, Tiffany Hink, Kimberly A. Reske, Kenneth Blount, Courtney Jones, Margaret H. Bost, Xiaoqing Sun, Carey-Ann D. Burnham, Erik R. Dubberke, Gautam Dantas, for the CDC Prevention Epicenter Program

**Affiliations:** 1 The Edison Family Center for Genome Sciences & Systems Biology, Washington University School of Medicine in St. Louis, St. Louis, MO 63110 USA; 2 Department of Pathology and Immunology, Division of Laboratory and Genomic Medicine, Washington University School of Medicine in St. Louis, St. Louis, MO 63110 USA; 3 Department of Medicine, Division of Infectious Diseases, Washington University School of Medicine in St. Louis, St. Louis, MO 63110 USA; 4 Rebiotix Inc. a Ferring Company, Minneapolis, MN 55113 USA; 5 Department of Biomedical Engineering, Washington University in St. Louis, St. Louis, MO 63110 USA; 6 Department of Molecular Microbiology, Washington University School of Medicine in St. Louis, St. Louis, MO 63110 USA

**Keywords:** Microbiota-based therapy, Placebo, Microbiome, Resistome, *Clostridioides difficile* infection, Antibiotic-resistant organisms

## Abstract

**Background:**

Intestinal microbiota restoration can be achieved by complementing a subject’s perturbed microbiota with that of a healthy donor. Recurrent *Clostridioides difficile* infection (rCDI) is one key application of such treatment. Another emerging application of interest is reducing antibiotic-resistant genes (ARGs) and organisms (AROs). In this study, we investigated fecal specimens from a multicenter, randomized, double-blind, placebo-controlled phase 2b study of microbiota-based investigational drug RBX2660. Patients were administered either placebo, 1 dose of RBX2660 and 1 placebo, or 2 doses of RBX2660 via enema and longitudinally tracked for changes in their microbiome and antibiotic resistome.

**Results:**

All patients exhibited significant recovery of gut microbiome diversity and a decrease of ARG relative abundance during the first 7 days post-treatment. However, the microbiome and resistome shifts toward average configurations from unperturbed individuals were more significant and longer-lasting in RBX2660 recipients compared to placebo. We quantified microbiome and resistome modification by RBX2660 using a novel “transplantation index” metric. We identified taxonomic and metabolic features distinguishing the baseline microbiome of non-transplanted patients and taxa specifically enriched during the process of transplantation. We elucidated the correlation between resistome and taxonomic transplantations and post-treatment dynamics of patient-specific and RBX2660-specific ARGs. Whole genome sequencing of AROs cultured from RBX2660 product and patient samples indicate ARO eradication in patients via RBX2660 administration, but also, to a lesser extent, introduction of RBX2660-derived AROs.

**Conclusions:**

Through shotgun metagenomic sequencing, we elucidated the effects of RBX2660 in the microbiome and resistome. Antibiotic discontinuation alone resulted in significant recovery of gut microbial diversity and reduced ARG relative abundance, but RBX2660 administration more rapidly and completely changed the composition of patients’ microbiome, resistome, and ARO colonization by transplanting RBX2660 microbiota into the recipients. Although ARGs and AROs were transmitted through RBX2660, the resistome post-RBX2660 more closely resembled that of the administered product—a proxy for the donor—than an antibiotic perturbed state.

**Trial registration:**

ClinicalTrials.gov, NCT02299570. Registered 19 November 2014

Video Abstract

## Background

Intestinal microbiota restoration by microbiota-based therapy, such as fecal microbiota transplantation (FMT) from healthy donors to patients, has been applied as a treatment for disorders caused by intestinal dysbiosis [1]. As the contributions of the gut microbiota to the host immune system, energy metabolism, and central nervous system have been uncovered, the range of potential applications of intestinal microbiota restoration therapy is expanding to various disorders, such as inflammatory bowel disease [2], functional gastrointestinal disorders [3], metabolic syndrome [4, 5], and neuropsychiatric disorders [6, 7]. Accordingly, studies for understanding and refining the action of intestinal microbiota restoration therapies are being actively conducted [8].

*Clostridioides difficile* infection (CDI) is one area where intestinal microbiota restoration therapy has been applied successfully. Although oral administration of antibiotics is the standard first-line therapy for CDI, antibiotics perturb the commensal gut microbiota and decrease colonization resistance against other pathogens [9, 10]. Approximately 15 to 30% of CDI patients therefore experience recurrent CDI (rCDI) resulting from either a relapse of the previous CDI or reinfection [11]. Moreover, antibiotic therapies during CDI treatment may promote the expansion of antibiotic-resistant organisms (AROs) such as vancomycin-resistant *Enterococci* (VRE) [12, 13]. On the other hand, intestinal microbiota restoration has shown to be effective for CDI treatment as well as the restoration of colonization resistance against *C*. *difficile* and AROs [14, 15]. Indeed, intestinal microbiota restoration has become a commonly performed investigational therapy for rCDI with decent success rates [8, 16–19].

However, due to the transmissive nature of the treatment, microbiota restoration therapy may communicate not only desirable but also undesirable factors derived from donors. For instance, the transmission of antibiotic-resistant genes (ARGs) and AROs derived from donor samples is a potential risk of fecal transplantation [20, 21]. AROs are responsible for increasing infection cases each year, and more than 35,000 patients died as a result of ARO infections in the United States in 2017 [22]. Recently, two cases of bacteremia caused by extended-spectrum beta-lactamase (ESBL)-producing *Escherichia coli* in patients after FMT from the same donor sample have been reported, resulting in the death of one of the patients [21]. Moreover, the dissemination of ARGs and pathogenic AROs in patients hampers effective medical care of infections and results in longer hospitalization and higher medical expenditures [23]. Still, multiple studies report efficient reduction of ARGs and decolonization of AROs through microbiota transplantation [24, 25].

**Fig. 1:**
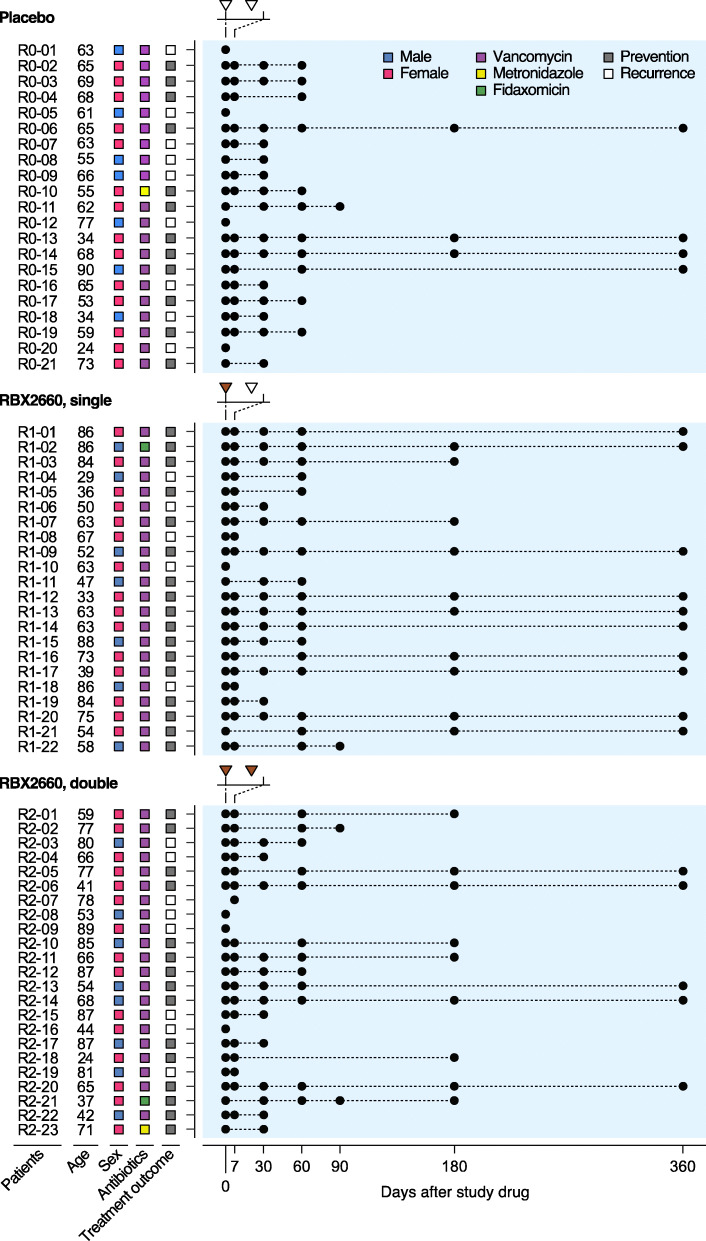
Study design for the use of RBX2660 to prevent recurrent *Clostridioides difficile* infection (rCDI). Total of 66 patients with a history of rCDI were treated with RBX2660 in a randomized and blinded manner. Placebo (white triangle) and RBX2660 (brown triangle) were administered and fecal samples (black circle) were collected at the indicated time points. Patients who were declared a new episode of rCDI within 60 days (white square) were moved to open-label treatment

In the current study, we explored the effect of a microbiota-based investigational drug RBX2660, a suspension of healthy donor microbiota [26–29], on the intestinal microbiome and resistome of recipients treated for rCDI. In an international, multicenter, randomized, and blinded phase 2b study, rCDI patients received either placebo (control group), one dose, or two doses of RBX2660 (Fig. 1), with more patients being recurrence-free after either RBX2660 regimen than placebo [26]. Through shotgun metagenomic sequencing, we demonstrate considerable shifts of taxonomic and resistome structures common to both placebo- and RBX2660-treated patients likely from discontinuation of antibiotics, particularly during the first week after treatment. By controlling for placebo effects, we could also distinguish taxonomic and resistome changes specific to RBX2660 treatment. Furthermore, we identified discriminative features strongly correlated with microbiota transplant and demonstrated an overall decrease in AROs as well as introduction of a few AROs by RBX2660.

## Results

### Study cohorts and sample collection

All donors of RBX2660 microbiota completed a comprehensive initial health and lifestyle questionnaire. Their blood and fecal samples were tested for immunodeficiency viruses, *C*. *difficile* toxin, and pathogens including AROs such as VRE and methicillin-resistant *Staphylococcus aureus* before enrollment into the donor program [27, 28]. Fecal specimens from a total of 66 patients and their corresponding RBX2660 products were collected during a multicenter, randomized, blinded, and placebo-controlled phase 2b study for the treatment of rCDI (Fig. 1) [26]. Ninety-four percent of all patients (62/66) had received vancomycin, with the remainder receiving metronidazole or fidaxomicin prior to study drug (Fig. 1). Twenty-one patients received 2 doses of placebo (14 females, 9 CDI recurrence, median age 63 years), 22 patients received 1 dose of RBX2660 and 1 dose of placebo sequentially (15 females, 5 CDI recurrence, median age 63 years), and 23 patients received 2 doses of RBX2660 (15 females, 8 CDI recurrence, median age 68 years) [26]. Each RBX2660 dose derives from a single donor, and RBX2660 dose selection was not constrained to ensure a single donor was represented in patients that received two RBX2660 doses (Supplementary Table 1). The first dose of study drug (RBX2660 or placebo) was administered 24–48 h following completion of antibiotic treatment for CDI, and the second treatment was administered 7 ± 3 days later (Fig. 1). Patients who experienced a new rCDI episode within 60 days after the first dose (9 placebo recipients, 5 single RBX2660 recipients, 8 double RBX2660 recipients) were moved to open-label treatment and received two additional doses of randomized RBX2660 (Fig. 1). Patient fecal specimens were collected at selected time points from baseline (day 0) through 365 days after the first dose. AROs from each fecal sample were isolated on selective media plates (the “Methods” section, Supplementary Table 2).

### RBX2660 shifted taxonomic structures of patients’ intestinal microbiome in a dose-dependent manner

**Fig. 2:**
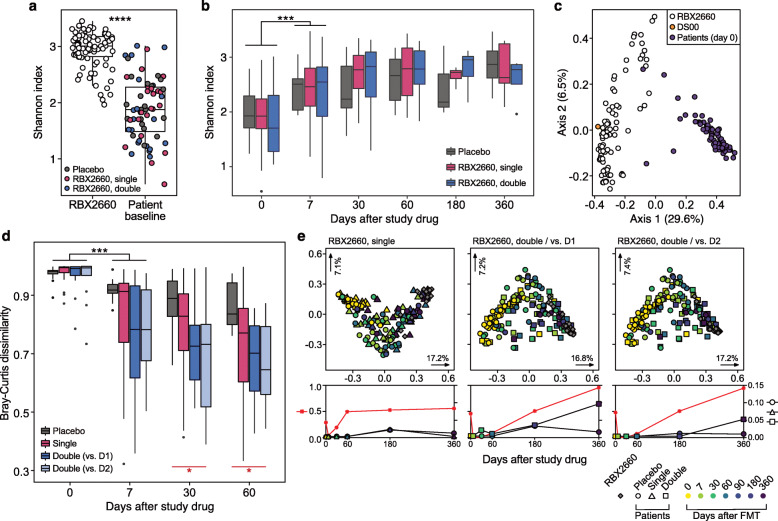
RBX2660 shifted taxonomic structures of the gut microbiome of recipients toward a healthy state. **a** RBX2660 products exhibited significantly higher alpha diversity than patient samples before treatment (Wilcoxon signed-rank test) based on the metagenomic taxonomic profiling data. **b** Alpha diversity of all patients including placebo recipients increased similarly after treatment. Changes in alpha diversity were significant for the first week after treatment, but there was no statistically significant difference among treatment groups (Kruskal-Wallis test). **c** Principal coordinates analysis (PCoA) showed a species-level clustering of RBX2660 (white) and pseudo-donor sample DS00 (yellow) distinct from patient baseline samples (violet). **d** Bray-Curtis distance between taxonomic structures of patients and corresponding RBX2660. D1 and D2 indicate the first dose and the second dose, respectively. DS00 was used for calculating the Bray-Curtis distance of placebo recipients. The decrease in Bray-Curtis distances was steepest during the first week after treatment (black, Wilcoxon signed-rank test). RBX2660 recipients showed a more dynamic decrease in Bray-Curtis distances than placebo recipients by day 60 (red, Kruskal-Wallis test). *P ≤ 0.05, **P ≤ 0.01, ***P ≤ 0.001, ****P ≤ 0.0001. **e** Upper panels: PCoA describing the direction of changes in taxonomic structures of RBX2660 recipients. Corresponding RBX2660 products and all placebo recipients were included. Lower panels: adjusted *P* values of PERMANOVA and relevant pairwise comparisons (Pillai-Bartlett non-parametric trace and Benjamini-Hochberg FDR correction). *P* values of comparisons between placebo and RBX2660 recipients (red asterisks, left *y*-axis), placebo recipients and RBX2660 (circle, right *y*-axis), single-dose recipients and RBX2660 (triangle, right *y*-axis), and double-dose recipients and RBX2660 (square, right *y*-axis) of PCoA plots were presented in corresponding lower panels

rCDI patients had significantly lower alpha diversity (Shannon diversity) than RBX2660 products before the treatment (Fig. 2a) as previously described with 16S sequencing [29]. Following study drug administration, the alpha diversity of all rCDI patients’ microbiota increased to near-RBX2660 levels regardless of the treatment group, with the steepest increase during the first week (Fig. 2b). The largest taxonomic structural shift also occurred during the first week in all treatment groups (Fig. S1 and S2).

Bray-Curtis dissimilarities between recipient and corresponding RBX2660 product were calculated to assess the level of taxonomic transformation toward that of RBX2660. For placebo recipients, the dissimilarity was measured from a pseudo-donor (DS00) profile calculated from the average species-level taxonomic profile of all RBX2660 products in this study (Fig. 2c). The mean Bray-Curtis dissimilarity of DS00 from RBX2660 products was 0.4926, which was lower than the inter-RBX2660 Bray-Curtis distance of 0.6274. Considering the thorough inspection criteria for donors of RBX2660 products, we defined RBX2660 microbiomes as “unperturbed” gut microbiomes. Bray-Curtis dissimilarities between patients and RBX2660 demonstrate that RBX2660 administration effectively changed recipients’ microbiome structure toward unperturbed configurations at a larger magnitude and for a longer duration as compared to placebo (Kruskal-Wallis test, *P* = 0.043 at day 30, *P* = 0.028 at day 60, Fig. 2d). These microbiome shifts by RBX2660 were not sensitive to the kind of antibiotic administered prior to RBX2660 (Fig. S3).

We further compared the original Bray-Curtis dissimilarities between patients and respective RBX2660 (*D*_R_) to dissimilarities between patients and other random RBX2660 (*D*_O_). RBX2660 recipients still exhibited lower *D*_O_s than those of placebo recipients in dose-dependent manner (Fig. S4), indicating that RBX2660 shifted patients’ gut microbiomes toward an unperturbed microbiome more actively than placebo. In addition, significantly lower *D*_R_s than *D*_O_s of double-dose recipients after the RBX2660 administration demonstrated dose-dependent and specific shifts toward corresponding RBX2660 (Fig. S4). Principal coordinates analysis (PCoA) and PERMANOVA for patients and RBX2660 also indicated that placebo recipients did exhibit taxonomic structural shifts toward RBX2660, but they were not as dramatic as those of double RBX2660 dose recipients toward the first dose RBX2660 (Fig. 2e).

When comparing groups based on rCDI treatment success, treatment-failure patients (who experienced a new rCDI episode within 60 days post-treatment) and treatment-success patients did not exhibit significant differences (Fig. S5a–c). This is likely due to limited number of treatment-failure samples after baseline, as patients were omitted from the current blinded study for the standard-of-care treatment at failure determination. Thus, we performed general linear model-based multivariate statistical analyses of patients’ baseline metagenomes using MaAsLin2 [30] to identify baseline features correlated to rCDI prevention success or failure. *Klebsiella pneumoniae* was the only species whose relative abundance was significantly associated with treatment failure in all patients (Fig. S5d). When patients were grouped by RBX2660 dose, the model identified *K*. *pneumoniae* as the only potential failure-associated feature again from placebo recipients (Fig. S5e) but did not from RBX2660 recipients.

### RBX2660 transplanted taxonomic structures to patients

To quantify and compare patients’ levels of change in microbiome composition, we calculated a transplantation index quantifying the extent of microbiome convergence toward corresponding RBX2660 product. This index was defined as the change in Bray-Curtis distances between baseline (Distance_BL_) and selected time point (Distance_*T*_), scaled by the distance from RBX2660 at baseline: (Distance_BL_ − Distance_*T*_)/Distance_BL_. DS00 was used for placebo recipients, who were then used to determine taxonomic transplantation success. To validate the transplantation index as a metric for quantifying microbiome shifts by RBX2660, we also calculated pseudo transplantation indices using dissimilarities between patients and random, non-corresponding RBX2660 products and compared them with the original transplantation indices. The dose-dependent increase in pseudo indices (Fig. S6) is additional evidence that RBX2660 shifted patients’ intestinal microbiome toward the unperturbed microbiome of RBX2660. Some of the pseudo indices were lower than zero, indicating that the transplantation index well reflects individual directionality of recipient’s microbiome shift toward respective RBX2660 (Fig. S6). Statistically significant differences between the original and pseudo transplantation indices of double-dose recipients, but not single dose (Fig. S6), connoted that double-dose administration allows more RBX2660-specific microbiome shift than single dose.

**Fig. 3:**
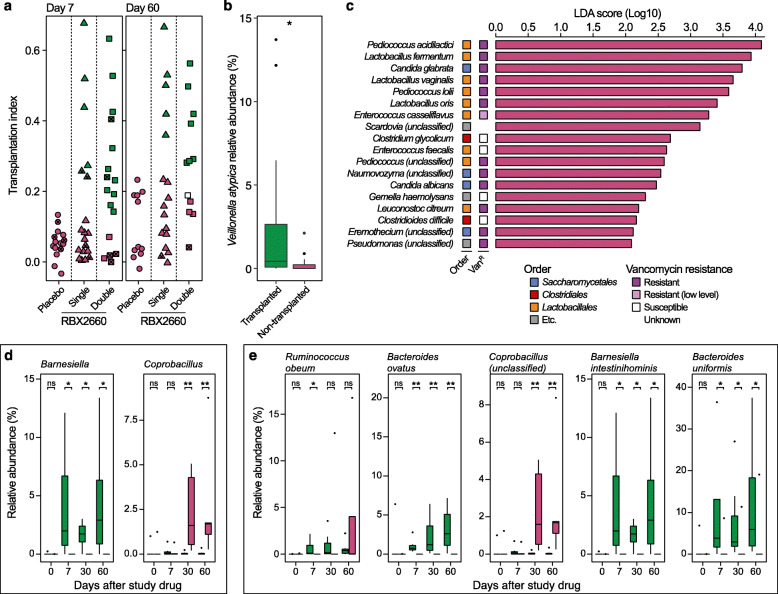
Discriminative taxonomic features of RBX2660 transplantation. **a** Transplantation index of patients on day 7 and 60. We defined the taxonomic transplantation as a state showing a higher transplantation index than that of all placebo recipients (green). The patients who were declared rCDI within 60 days were marked (x). The white square represents the patient who exhibited a lower transplantation index for the first dose but a higher transplantation index for the second dose than placebo patients (R2-21, Fig. S7a). **b** Higher baseline relative abundances of *Veillonella atypica* in patients who showed durable taxonomic transplantation by day 60 in both single and double RBX2660 treatment groups (Wilcoxon signed-rank test, *P* = 0.027). **c** Linear discriminant analysis effect size (LEfSe) determined baseline taxonomic features of the obstinate non-transplanted patients who exhibited lower transplantation indices than placebo recipients at day 60 after double RBX2660 treatment. Thirteen species among 18 taxonomic features were intrinsically vancomycin resistant (violet square, including *E*. *casseliflavus* of low resistance). There was no taxonomic feature specific to transplanted patients determined by LEfSe. Genus (**d**) and species enrichment (**e**) associated with the taxonomic transplantation (transplanted, green; non-transplanted, purple) were identified through a two-part zero-inflated Beta regression model with random effects (ZIBR) test. *P ≤ 0.05, **P ≤ 0.01

RBX2660 recipients were categorized as transplanted or non-transplanted based on whether their transplantation index was higher (transplanted) or lower (non-transplanted) than the maximum value of the placebo group (Fig. 3a). The transplantation ratio trended higher in double-dose recipients versus single-dose recipients; this categorization showed 33.3% and 70.6% transplantation for single- and double-dose recipients, respectively, by day 7 (Chi-square test, *P* = 0.02752), and 29.4% and 58.3% by day 60 (Chi-square test, *P* = 0.1212). Non-transplanted patients at day 7 maintained non-transplanted status until day 60, regardless of dose. On the other hand, 1 single-dose recipient (R1-21) and 3 double-dose recipients (R2-01, R2-03, and R2-14) failed to maintain their transplanted state at day 7 until day 60 and eventually reverted to below the transplantation threshold. *Veillonella atypica* was the only baseline taxonomic feature determined by linear discriminant analysis effect size (LEfSe) [31] that distinguished patients with successful microbiome transplantation by day 60 from non-transplanted patients in both single and double RBX2660 treatment arms (Fig. 3b).

Although double RBX2660 dosage led to more effective transplantation of RBX2660 microbiome structure, there were 4 double-dose recipients (R2-01, R2-02, R2-03, R2-14) who showed lower transplantation indices than placebo recipients at day 60 (Fig. 3a and S7a). All of the 4 patients received vancomycin prior to RBX2660 administration (Fig. 1). We determined 18 taxa (Fig. 3c) and 21 functions (Fig. S7b) as features specifically explaining the baseline microbiome of these 4 patients by comparing with other double-dose recipients that showed durable taxonomic transplantation by day 60 using LEfSe [31]. Of these, 4 taxonomic features were fungi, which are intrinsically vancomycin insensitive, and 7 functional features of eukaryote-specific metabolic pathways (Fig. 3c and S6b). We further investigated the predicted vancomycin insensitivity of other taxonomic features and found 8 additional intrinsically vancomycin-resistant bacteria including *Pediococcus* strains [32–34] and *Lactobacillus* and *Leuconostoc* strains [35–37] as well as gram-negative and fungal strains. *Enterococcus casseliflavus*, which has low level resistance to vancomycin, was also identified [38]. Four taxa (*Clostridium glycolicum* [39], *Gemella haemolysans* [40], *E*. *faecalis* [41], and *C*. *difficile* [42]) are predicted to be vancomycin susceptible. Compared to the transplanted patients, the 4 non-transplanted patients did not exhibit any other distinctive taxonomic characteristics in terms of alpha diversity and composition of *Bacteroidetes*, *Firmicutes*, and *Proteobacteria* phyla (Fig. S7c–g).

Beyond baseline features, we further investigated which taxa were enriched during the process of transplantation. Through a two-part zero-inflated beta regression model with random effects (ZIBR) test [43], we investigated a subset of 12 patients (R1-02, R1-03, R1-09, R1-14, R1-21, R2-05, R2-06, R2-10, R2-11, R2-12, R2-13, and R2-20) matched for 4 different time points: baseline, day 7, 30, and 60. ZIBR models a taxon’s presence and absence (logistic component) as well as its non-zero abundance (Beta component), while incorporating patient and time as random variables (random intercepts). Only two genera, *Barnesiella* and *Coprobacillus*, are significantly correlated with the taxonomic transplantation. *Barnesiella* is significantly overrepresented in the transplanted patients as early on as day 7, while *Coprobacillus* is overrepresented in non-transplanted patients at days 30 and 60 (Fig. 3d). At the species level, ZIBR models identified *Barnesiella intestinihominis*, *Coprobacillus* (unclassified), *Bacteroides ovatus*, *Bacteroides uniformis*, *Ruminococcus obeum*, and *Akkermansia muciniphila* (Fig. 3e, *A*. *muciniphila* was omitted because its time point comparisons were not statistically significant in the actual data). *Barnesiella intestinihominis* and unclassified *Coprobacillus* species followed near-identical patterns from the genus-level analysis due to single species being identified from each genus.

### Resistome regression significantly correlated with transplantation index

**Fig. 4:**
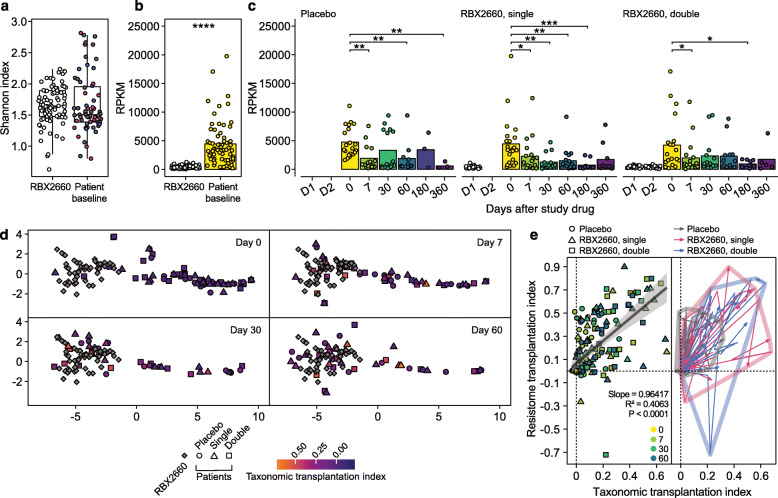
RBX2660 fluctuated resistome structures of patients via the taxonomic transplantation. **a** Alpha diversity of baseline patient resistomes was comparable to that of RBX2660 (*P* = 0.18). **b** However, baseline patient resistomes had a greater antibiotic-resistant gene (ARG) reads per kilobase per million sample reads (RPKM, Wilcoxon signed-rank test). **c** Significant decrease in ARG RPKM was observed over time in all treatment groups (Wilcoxon signed-rank test with Benjamini-Hochberg FDR correction, FDR < 0.05). Bars indicate mean of individual ARG relative abundances. D1, the first dose; D2, the second dose. **d** Patients and RBX2660 products were clustered separately in *t*-distributed stochastic neighbor embedding (t-SNE) analysis of resistome structures at day 0. Patient resistome became similar to RBX2660 over time, but the speed of change varied for each patient regardless of RBX2660 dose and taxonomic transplantation index. **e** RBX2660 simultaneously fluctuated both taxonomic and resistome structures more dynamically as compared to placebo. *P ≤ 0.05, **P ≤ 0.01, ***P ≤ 0.001, ****P ≤ 0.0001

Prior to treatment, rCDI patients showed a similar resistome alpha diversity (Wilcoxon signed-rank test, *P* = 0.18, Fig. 4a) when ARGs were grouped into ARG families based on the organizational structure in CARD [44]. However, the relative abundance of total ARGs was significantly higher in the patients than RBX2660 (Wilcoxon signed-rank test, *P* < 0.0001, Fig. 4b). It decreased over time in all treatment arms including the placebo group (Fig. 4c). Patients’ resistome composition was distinct from RBX2660 products, but the antibiotic treatment prior to study drug administration did not lead to noticeable difference in resistome (Fig. S8a–c). Specifically, major facilitator superfamily (MFS) and resistance-nodulation-cell division (RND) efflux pumps were the major ARG families present in rCDI patients before the treatment, whereas CfxA beta-lactamase, tetracycline-resistant ribosomal protection proteins, and Erm 23S rRNA methyltransferases were representative of the RBX2660 resistome (Fig. Se).

We tracked individual changes in resistome composition of each patient for 60 days using *t*-distributed stochastic neighbor embedding (t-SNE) analysis [45] and resistome transplantation indices defined analogously to the microbiome transplantation index. rCDI patients showed distinctive resistome compositions as compared to those of RBX2660 prior to the treatment, but over time their resistome compositions converged to become similar to RBX2660 (Fig. 4d). The speed of resistome transformation toward RBX2660-like structures varied by patient. The convergence toward RBX2660 resistome structure showed strong correlation to the taxonomic transplantation irrespective of treatment arm (*R*^2^ = 0.406, *P* < 0.0001, Fig. 4e). RBX2660 administration led to higher taxonomic and resistome transplantation indices than the placebo (Fig. 4e).

**Fig. 5:**
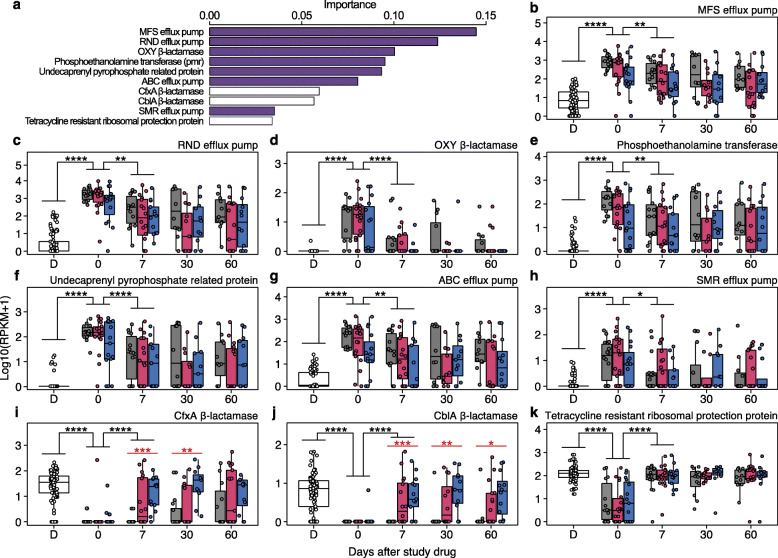
Recipients adopted a resistome profile similar to that of donors. **a** Ten most important patient-specific (violet) and RBX2660-specific (white) antibiotic-resistant gene (ARG) families were identified through random forest classifier. **b**–**k** Relative abundance of the selected 10 ARGs in RBX2660 (D) and patients who received placebo (gray), single RBX2660 (red), and double RBX2660 (blue). Relative abundance of patient-specific ARGs decreased over time in all patients without statistically significant difference among treatment arms (**b**–**h**). Relative abundance of the two RBX2660-specific beta-lactamases in patients increased by RBX2660 administration in a dose-dependent manner (**i**–**j**, red, Kruskal-Wallis test). Tetracycline-resistant ribosomal protection protein was a RBX2660-specific ARG, but its relative abundance in placebo recipients also increased after the treatment (**k**). These changes were significant during the first week after the treatment (black, Wilcoxon signed-rank test). *P ≤ 0.05, **P ≤ 0.01, ***P ≤ 0.001, ****P ≤ 0.0001

To identify features distinguishing patient and RBX2660 resistomes, we used a random forest classifier (Fig. S9a–b). Of the top 10 features of importance, 7 ARGs, namely MFS efflux pump, RND efflux pump, OXY β-lactamase, Pmr phosphoethanolamine transferase, undecaprenyl pyrophosphate related proteins, ATP-binding cassette (ABC) efflux pump, small multidrug resistance (SMR) efflux pump, and tetracycline-resistant ribosomal protein, were specific to patients’ baseline resistomes. Class A β-lactamases (CfxA and CblA) and a tetracycline-resistance protein, which are frequently identified in healthy populations or donor stools in FMT trials [20, 46–49], were classified as RBX2660-specific ARGs (Fig. 5a). Relative abundances of all selected ARGs were significantly altered in recipients one week after study drug administration (Fig. 5b–k). The regression of patient-origin ARGs occurred in all patients without statistically significant differences among placebo and RBX2660 recipients (Fig. 5b–h and S9c–i). Administration of RBX2660 increased relative abundances of RBX2660-origin β-lactamases in a dose-dependent manner (Fig. 5i, j), while the relative abundance of tetracycline-resistant ribosomal protection protein increased in all patients irrespective of treatment (Fig. 5k).

### RBX2660 effectively cleared AROs compared to placebo but introduced new AROs

**Fig. 6:**
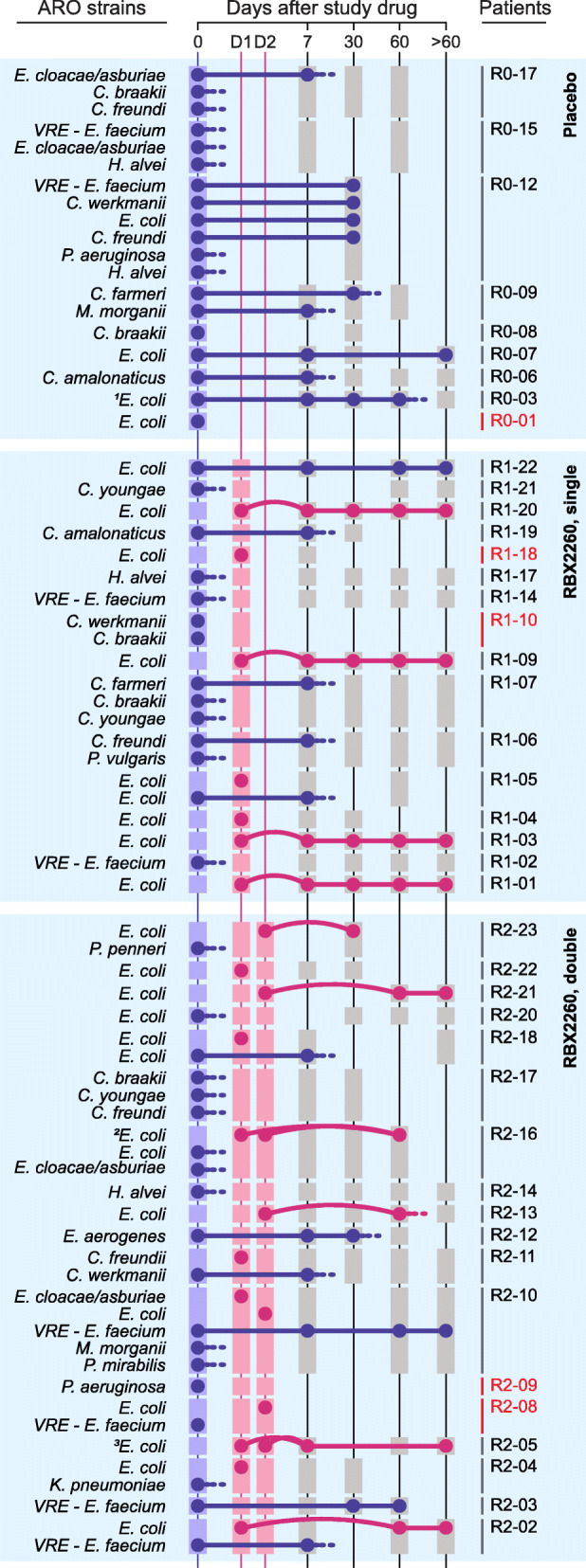
RBX2660 effectively cleared antibiotic-resistant organisms (AROs) compared to placebo and simultaneously introduced new AROs. We specifically tracked patient-derived (blue dot) and RBX2660-derived AROs (red dot). Patients with no ARO detected from both the baseline sample and corresponding RBX2660 were excluded. Persistency (solid line), disappearance (dash line), and introduction (curved line) of the AROs were determined by genomic comparison of AROs (the “ARO tracking and SNP calling” section). Squares indicate the sample availability (blue, patient baseline samples; red, RBX2660; gray, patient samples after RBX2660 administration). Patients with no samples after day 7 were marked with red. ^1^R0-03 showed 2–3 separate lineages of *E*. *coli* prior to day 30, which were reduced to 1 lineage by day 60. ^2^Patient R2-16 received the same RBX2660 product twice. ^3^Although the two RBX2660 products for patient R2-05 were prepared from different donor samples, ARO *E*. *coli* strains screened from those appeared to be clonal (distance = 8 SNPs)

We identified both persisting and newly introduced AROs based on whole genome sequence analyses of isolates from both blind and open-label treatment patients. ARO isolates were *Escherichia coli* (*n* = 104), vancomycin-resistant *Enterococcus* (VRE) (*n* = 25), and other species (*n* = 135). The majority of RBX2660-derived AROs were *E*. *coli* (Fig. 6). We selected *E*. *coli* and VRE, the plurality of screened AROs, for further analyses based on availability of donor-recipient matched pairs and longitudinal samples. Pairwise average nucleotide identity (ANI) was above 97% for all *E*. *coli* isolates (Fig. S10), with more than 99.43% identity for all VRE (Fig. S11). Core genome phylogeny indicated the *E*. *coli* were mostly of the B2 and D phylogroups. Isolates not only clustered together based on the patient of origin, but also with their corresponding RBX2660 (Fig. S10).

In general, RBX2660 recipients demonstrated faster clearance of AROs as compared to placebo recipients (Fig. 6). Simultaneously, new AROs from RBX2660, mostly *E*. *coli*, were introduced to corresponding patients. Calculation of single nucleotide polymorphism (SNP) distances (the “ARO tracking and SNP calling” section) revealed many of these AROs were likely clonal, with a median of 6 SNPs for all pairwise distances indicating near-identical genomes (Supplementary Table 3). We sorted post-treatment ARO *E*. *coli* into RBX2660-origin or patient-origin strains and determined clonal persistence following RBX2660 intervention. The introduced AROs were found in patients longitudinally for up to 1 year post-treatment (Fig. 6). In some cases, we observed clonal persistence of patient AROs (e.g., patients R1-05 and R2-18), while in some we observed strain replacement by RBX2660-derived AROs (*e*.*g*., patient R2-16). Interestingly, patients receiving the same RBX2660 product did not display identical trends. Patient R2-21 received the same RBX2660 product as R2-18 yet only R2-21 engrafted the RBX2660 ARO (Fig. 6). Persisting AROs derived from patients R1-05 and R2-18 showed higher phenotypic resistance than their corresponding RBX2660-derived AROs, which failed to engraft. On the other hand, patient R2-21 lacked baseline AROs and perhaps provided a “clean slate” for the ARO engraftment.

Isolate ARGs did not indicate a changing resistance profile for these ARO lineages over time. For instance, *E*. *coli* isolates exhibited an average of 60 predicted ARGs, and these numbers remained stable throughout the time frame of this investigation (Supplementary Table 4). The 15 RBX2660-origin AROs which were engrafted to corresponding recipients harbored beta-lactamase genes such as AmpC (12 AROs), TEM-1 (8), CARB (3, one each of CARB-17, 19, and 20), or CTX-M-14 (1) (Supplementary Table 4). Antibiotic susceptibility testing (AST) corroborated these findings on the phenotypic level with all introduced AROs being resistant to ciprofloxacin and levofloxacin, and 60% (9/15) resistant to ampicillin (Fig. S12). Approximately half were resistant or intermediate to trimethoprim-sulfamethoxazole (7) and doxycycline (7), and a few were resistant to ampicillin-sulbactam (3) and cefazolin (4), while all were susceptible to cefotetan, ceftazidime, meropenem, imipenem, piperacillin-tazobactam, ceftazidime-avibactam, amikacin, aztreonam, tigecycline, and nitrofurantoin (Supplementary Table 2). The introduced AROs were *Enterobacteriaceae* and resistant to a median of 4 antibiotics, which was less than that of the patient-origin *Enterobacteriaceae* AROs (median resistance to 7 antibiotics, Supplementary Table 2). The most resistant isolate introduced from RBX2660 was an *E*. *coli* strain which was engrafted into patient R1-09. It was retrieved at 5 subsequent time points (final fecal sample collected at 12 months, all < 20 SNPs, Fig. 6, Supplementary Table 2). This isolate, DI11, was resistant to ceftriaxone and cefepime and classified as an ESBL-producing *E*. *coli* (Supplementary Table 2). We further validated ESBL production of DI11 and the corresponding patient isolates using double-disk diffusion tests (Supplementary Table 6).

## Discussion

We investigated factors underlying changes in the microbiome derived from RBX2660 in a randomized, double-blind, placebo-controlled clinical trial [26]. Consistent with a previous evaluation [29] but in higher resolution using shotgun metagenomic sequencing, we demonstrated RBX2660 dose-dependent changes in the microbiome. Still, all patients initially increased alpha diversity and shifted taxonomic structure regardless of treatment, which could be accredited to the natural trajectory of recovery after antibiotic discontinuation [10, 50]. We hypothesized that it would be possible to distinguish RBX2660-derived effects from the microbiome recovery after antibiotic discontinuation by assessing both extent and direction of microbiome shifts of placebo recipients as thresholds. To test the hypothesis, we developed a simple yet novel metric, the transplantation index. The transplantation index accounts for long-term changes in the microbiome toward corresponding RBX2660 while controlling for individual variation in baseline composition. With the highest transplantation index among placebo recipients as threshold, we demonstrated that RBX2660 recipients exhibited stronger and longer-lasting microbiome changes toward corresponding RBX2660 than placebo recipients.

In an effort to predict transplantation success, we identified baseline taxonomic features that had strong correlations with taxonomic non-transplantation. Species with intrinsic vancomycin resistance were discriminative baseline features of the 4 patients who failed to acquire or maintain transplantation by double RBX2660 administration by day 60 (R2-01, R2-02, R2-03, and R2-14). Previously reported microbiome signatures of vancomycin administration including lower diversity, lower *Firmicutes*, and higher *Proteobacteria* levels [10, 51, 52] could not distinguish the 4 non-transplanted patients from transplanted patients. The specific enrichment of intrinsically vancomycin-resistant species therefore could be an indicator of more severe microbiome disturbance by vancomycin. Interestingly, the baseline relative abundance of *V*. *atypica* was significantly and positively correlated with durable taxonomic transplantation of RBX2660 microbiome in both the single- and double-dose arms. *V*. *atypica* has long been known as an oral bacteria that communicates and develops oral plaque biofilm with lactic acid bacteria [53, 54], but a recent study has highlighted its capacity to build metabolomic networks via a peculiar metabolic function—converting lactate to propionate—in the host gut [55]. Further studies combining both metagenomic and metabolomic analyses are required to uncover the mechanism underlying the positive role of *V*. *atypica* in durable microbiota transplantation. Relative abundances of *Barnesiella* and *Coprobacillus* genera are significantly correlated with taxonomic transplantation status. *Barnesiella*, which exhibited positive correlation with taxonomic transplantation, also has been linked to clearance of VRE colonization in mice [56]. Two *Bacteroides* species, *B*. *ovatus* and *B*. *uniformis*, were overrepresented in transplanted patients, reflecting the previous report on their correlation with the unperturbed gut microbiome [57, 58].

We also hypothesized that microbiome features of patients are also associated with the prevention of CDI recurrence during the RBX2660 clinical trial. General linear model-based multivariate statistical analyses identified *K*. *pneumoniae* as a species associated with treatment failure from all patients or only placebo recipients but did not from RBX2660 recipients. Baseline *K*. *pneumoniae* might indeed be a rCDI-associated feature, such as a biomarker of the imbalanced microbiome [59] that underlies CDI, but not correlate with the outcomes of RBX2660 recipients whose microbiomes were affected by RBX2660. Together with the higher efficacy for RBX2660 on the rCDI prevention than placebo [26], the model outputs suggest that RBX2660 transplantation restored the disturbed intestinal microbiota to outcompete *C*. *difficile*. We reckoned that both dose levels provide enough unperturbed microbiota to exceed a minimum threshold to achieve clinical efficacy, and the second dose provides additional microbiota from which the taxonomic transplantation may arise. Despite their apparent difference between transplantation indices of single- and double-dose recipients, the two treatment arms showed equivalent clinical efficacy [26]. Likewise, although early-stage transplantation by day 7 appeared to be an important factor determining durable transplant by day 60, it did not always secure successful prevention of rCDI and vice versa.

The differences between rCDI patients and RBX2660 in both ARG relative abundance and resistome architecture became narrowed in all the three treatment arms over time. These outcomes suggest that antibiotic discontinuation could be the driver of the changes in resistome during this clinical trial. Despite the natural recovery after antibiotic discontinuation, we hypothesized that transplantation of RBX2660 microbiota shaped patient resistome. RBX2660 indeed simultaneously introduced and eradicated both ARGs and AROs in patients during the process of transplantation. Previous studies have also demonstrated the efficacy of FMT for eradicating AROs [60], but to our knowledge this is the first to comprehensively track clonality for both RBX2660- and patient-derived ARO isolates. Most introduced AROs were antibiotic-resistant *E*. *coli* that are commonly present in a healthy population [61, 62].

We identified one ESBL-producing *E*. *coli* strain from a RBX2660 product carrying AmpC and CTX-M-14, whose RBX2660 product was administered to one patient, R1-09. The patient was a single-dose recipient, with recorded treatment success (i.e., no recurrence of CDI and absence of diarrhea for 8 weeks post-treatment) and no known clinical disease resulted from the trial. ESBL-producing *E*. *coli* are not inherently more virulent than other strains but can pose a therapeutic challenge if infection occurs [63]. Of note, this trial enrolled patients from December 2014 to November 2015, prior to recognition of ESBL as an important aspect of donor screening. At that time, donor stools were screened for carbapenem-resistant *Enterobacteriaceae* (CRE) but not ESBL, whereas Rebiotix now screens all donor stools for both CRE and ESBL. Moreover, to date, there have been no adverse infection events due to bacterial transmission from RBX2660 in any clinical trials. In light of a recent death caused by ESBL-producing *E*. *coli* bacteremia in an immunocompromised patient after FMT [21], our findings highlight the importance of a controlled and regulated donor screening program as well as mandatory, monitored safety reporting. Likewise, our findings prompt a general consideration of risk factors for infections from intestinal microorganisms in any life biotherapeutic investigational product.

## Conclusions

We thoroughly examined the impact of RBX2660 on the taxonomic structure, resistome, and ARO colonization of recipients during a randomized and placebo-controlled clinical trial. This study is based on samples from a completed placebo-controlled clinical trial of intestinal microbiota restoration, which enabled us to determine microbiome effects of the microbiota-based drug. Using the transplantation index, the current study demonstrated that RBX2660 administration transplanted its microbiota in the recipients in a dose-dependent manner. *V*. *atypica*- and intrinsic vancomycin-resistant species were discriminative features of patients showing long-lasting microbiota transplantation and resisting microbiota transplantation, respectively. While antibiotic discontinuation alone significantly reduced patient-origin ARGs, RBX2660 administration led to more dynamic transformations of the resistome. RBX2660 simultaneously introduced RBX2660-origin ARGs in a dose-dependent manner. RBX2660 more efficiently decolonized AROs than placebo but simultaneously introduced new AROs. Genomic outcomes of intestinal microbiota restoration with RBX2660 in the current study show both latent limitations of microbiota transplantation as well as its potential benefits and highlight the importance of the design and quality control of microbiota-based drugs.

## Methods

### Study cohort, drug, and specimen

Subjects were recruited from among 17 centers in the USA and Canada from 10 December 2014 through 13 November 2015. Subjects were adults with recurrent CDI who have had either (i) at least two recurrences after a primary episode (total three CDI episodes) and had completed at least two rounds of oral antibiotic therapy or (ii) had at least two episodes of severe CDI resulting in hospitalization. They were randomly assigned to one of three treatment groups: placebo, single, or double doses of RBX2660. All treatments were blinded and delivered by enema [26]. The second dose was administered approximately 7 days after the first dose. For patients that received two RBX2660 doses, donor selection was random and not constrained to provide a single representative donor per patient.

The selection and screening of donors for RBX2660 were performed as previously described [27, 28]. The placebo composed of normal saline and formulation solution including cryoprotectant in the same proportions used for the RBX2660 preparation. RBX2660 and placebo were stored frozen after preparation until administration. They were thawed for 24 h in a refrigerator and administered within 48 h after thawing. AROs were isolated from patient fecal samples and RBX2660 products on selective agar media plates, chromID VRE (bioMerieux, Marcy-l’Etoile, France), MacConkey with Cefotaxime (Hardy Diagnostics, Santa Maria, CA), MacConkey with Ciprofloxacin, (Hardy Diagnostics), and HardyCHROM^TM^ ESBL (Hardy Diagnostics), at 35°C in air. The remaining fecal samples were stored frozen at − 80 °C until metagenomic DNA extraction. Isolate colonies were sub-cultured to trypticase soy agar with 5% sheep blood (Becton Dickinson, Franklin Lakes, NJ) and identified using VITEK MS matrix-assisted laser desorption/ionization time-of-flight mass spectrometry (MALDI-TOF MS) system [64, 65]. Each isolate was frozen in tryptic soy broth with glycerol at − 80°C.

### Antibiotic susceptibility testing

Antibiotic susceptibility testing was performed through Kirby Bauer disk diffusion, and the resulting zone sizes were interpreted according to the M100 document from the Clinical and Laboratory Standards Institute [66].

### DNA extraction and sequencing

Metagenomic DNA was extracted from approximately 100 mg of fecal samples using DNeasy PowerSoil Kit (Qiagen) following the manufacturer’s protocol excepting the lysis step: fecal samples were lysed by 2 rounds of bead beating for 2 min (total 4 min) at 2500 oscillations/min using a Mini-Beadbeater-24 (Biospec Products). Samples were chilled on ice for 2 min between the two bead beating rounds. Extracted DNA was quantified using a Qubit fluorometer dsDNA HS Assay (Invitrogen) and stored at − 20 °C until the library preparation. Metagenomic DNA was diluted to 0.5 ng/μL before preparing the sequencing library. Libraries were prepared using the Nextera DNA Library Prep Kit (Illumina) as previously described [67]. The libraries then were purified through the Agencourt AMPure XP system (Beckman Coulter) and quantified by Quant-iT PicoGreen dsDNA Assay Kit (Invitrogen) before sequencing. Approximately 70 library samples were pooled in an equimolar manner at the final concentration of 5 nM for each sequencing lane. Prepared pools were submitted for 2 × 150 bp paired-end sequencing on an Illumina NextSeq High-Output platform at the Center for Genome Sciences and Systems Biology at Washington University in St. Louis with a target sequencing depth of approximately 5.5 million reads per sample.

Isolate genomic DNA was extracted using QIAmp BiOstic Bacteremia DNA Kit (Qiagen). Libraries for whole genome sequencing of isolates were prepared from diluted genomic DNA (0.5 ng/μL) as described above. About 180 libraries were pooled together in an equimolar manner at the final concentration of 5 nM for each sequencing lane. Prepared pools were submitted for 2 × 150 bp paired-end sequencing on an Illumina NextSeq High-Output platform at the Center for Genome Sciences and Systems Biology at Washington University in St. Louis with a target sequencing depth of approximately 2 million reads per sample.

### Data processing and genome assembly

Sequence reads were binned by index sequence. Adapter and index sequences were trimmed using Trimmomatic v.0.38 [68] using the following parameters: java -Xms2048m -Xmx2048m -jar trimmomatic-0.38.jar PE -phred33 ILLUMINACLIP: NexteraPE-PE.fa:2:30:10:1:true SLIDINGWINDOW:4:15 LEADING:10 TRAILING:10 MINLEN:60. Human sequence contamination was eliminated using Deconseq [69], and the qualities of resulting reads were verified by FastQC (https://github.com/s-andrews/FastQC).

Isolate genomes were assembled, assessed, and annotated using SPAdes [70], QUAST [71], and Prokka [72], respectively. Average nucleotide identity between *E*. *coli* and VRE isolate pairs were calculated using dnadiff [73]. Within-species pan genomes and core genome alignments were obtained with Roary [74] with default parameters, using 24 and 4 NCBI reference strains (Supplementary Table 5) for *E*. *coli* and VRE, respectively, with additional *Escherichia fergusonii* and general *Enterobacter faecalis* as outgroups. Alignments were converted via FastTree [75] and visualized on iTOL v4 [76].

### Microbiomic analyses

Microbiome taxonomic composition was predicted by MetaPhlAn v2.0 [77] and controlled for relative abundance. Genus-level composition plots were obtained by grouping together genus present in less than 50% of samples as “Other.” DS00 pseudo-donor microbiome was obtained by averaging the species-level taxonomic profiles of all RBX2660 microbiomes. Bray-Curtis distances were calculated using the vegan package [78] and visualized as PCoA plots via the ape package [79] in R 3.5.3. LEfSe [31] identified baseline taxonomic and metabolic features distinguishing transplanted and non-transplanted patients (alpha value for the factorial Kruskal-Wallis test = 0.05, threshold on the logarithmic LDA score = 2). HUMAnN2 [80] was employed for metabolic pathway prediction. Longitudinal changes distinguishing transplanted and non-transplanted patients were identified using the ZIBR [43] package in R. Taxa were filtered for non-zero presence in at least 40% samples, and > 0.01 relative abundance in the 90th percentile. Each taxon’s relative abundance was modeled as both the logistic (*X*) and beta (*Z*) components (alpha value for Benjamini-Hochberg-adjusted *P* = 0.05) with transplantation outcome as a fixed effect. Baseline features distinguishing patients with and without rCDI were detected using MaAsLin2. MaAsLin2 is a general linear model-based association detector for microbiome associations with metadata, in this case associations with treatment outcome (success or failure). Taxa were filtered with a minimum prevalence of 0.1 and a minimum relative abundance of 0.0001. Five different models were fitted: one for all patients (total *n* = 63), one for each treatment arm separately (placebo, *n* = 21; single dose, *n* = 22; double dose, *n* = 21), as well as one for RBX2660 recipients (*n* = 43) (alpha value for Benjamini-Hochberg-adjusted *P* = 0.05).

### Resistome identification and random forest classifier

ARGs in the microbiome were identified using ShortBRED [81] with CARD [44]. Isolate ARGs were identified with RGI and CARD [44, 82]. The resulting genes were manually curated into more general ARG families (*n* = 64). A subset of 70% of available resistomes were then used to train a random forest classifier distinguishing patient baseline and RBX2660 resistomes (training set *n* = 103), which was then tested on the remaining samples (test set *n* = 45). The random forest classifier was built with the package scikit-learn (https://scikit-learn.org/stable/index.html) on Python 3.7.3, with trees averaging 12 nodes and a maximum depth of 4.

### ARO tracking and SNP calling

SNPs were called using Bowtie2 [83], SAMtools, and BCFtools [84], with the first isolate from the patient or corresponding RBX2660 product used as the reference genome. Reads from subsequent isolates of the same species were aligned against the reference with Bowtie2 (-X 2000 --no-mixed --very-sensitive --n-ceil 0,0.01). BAM files were obtained and sorted with SAMtools (view and sort), which were then converted to pileup files (mpileup). BCFtools view generated VCF files, and variants were called, with the following criteria: minimum coverage of 10 reads per SNP, major allele frequency above 95%, and FQ-score of − 85 or less. Indels were excluded. VCF files for each patient were compiled with BCFtools merge, after which SNPs were parsed and counted using custom python and R scripts.

## Supplementary information

**Additional file 1: Figure S1.** Taxonomic overview of patient stool samples at the genus level. **Figure S2.** Taxonomic shift by treatments (related Fig. 2). **Figure S3.** The effect of antibiotics prior to study drug on taxonomic shift by RBX2660 (related Fig. 2 and 3). **Figure S4.** Bray-Curtis dissimilarities between patients and respective RBX2660 (*D*_R_) or other random RBX2660 (*D*_O_). **Figure S5.** Changes in the Bray-Curtis dissimilarities between a patient and corresponding donor. **Figure S6.** Transplantation indices (TIs) and pseudo transplantation indices (pTIs). **Figure S7.** Additional discriminative features of the obstinate patients (related Fig. 3). **Figure S8.** Comparison of resistome compositions. **Figure S9.** Random forest classifier successfully distinguished between donor and patient baseline resistomes (related Fig. 5). **Figure S10.** Average nucleotide identity (ANI) and core genome phylogeny of *E. coli* isolates. **Figure S11.** Average nucleotide identity (ANI) and core genome phylogeny of VRE isolates. **Figure S12.** Antibiotic susceptibility testing (AST).

**Additional file 2.** Supplementary Table 1 Patient drug identifiers.

**Additional file 3.** Supplementary Table 2 AST results.

**Additional file 4.** Supplementary Table 3 SNP distances.

**Additional file 5.** Supplementary Table 4 ARG data.

**Additional file 6.** Supplementary Table 5 NCBI references.

**Additional file 7.** Supplementary Table 6 Double disk test.

## Data Availability

The metagenomic sequencing data are uploaded to NCBI under BioProject PRJNA606075 (https://www.ncbi.nlm.nih.gov/bioproject/606075). The isolate genome sequences and assemblies are uploaded to NCBI under BioProject PRJNA606074 (https://www.ncbi.nlm.nih.gov/bioproject/606074).

## Abbreviations

FMT: Fecal microbiota transplantation
ARG: Antibiotic-resistant gene
ARO: Antibiotic-resistant organism
CDI: *C*. *difficile* infection
rCDI: Recurrent *C*. *difficile* infection
VRE: Vancomycin-resistant *Enterobacter*
SNP: Single nucleotide polymorphism
ANI: Average nucleotide identity

## References

[1] Smits WK, Lyras D, Lacy DB, Wilcox MH, Kuijper EJ (2016). *Clostridium difficile* infection. Nat Rev Dis Primers.

[2] Colman RJ, Rubin DT (2014). Fecal microbiota transplantation as therapy for inflammatory bowel disease: a systematic review and meta-analysis. J Crohns Colitis.

[3] Pinn DM, Aroniadis OC, Brandt LJ (2015). Is fecal microbiota transplantation (FMT) an effective treatment for patients with functional gastrointestinal disorders (FGID)?. Neurogastroenterol Motil.

[4] Leshem A, Horesh N, Elinav E (2019). Fecal microbial transplantation and its potential application in cardiometabolic syndrome. Front Immunol.

[5] de Groot PF, Frissen MN, de Clercq NC, Nieuwdorp M (2017). Fecal microbiota transplantation in metabolic syndrome: history, present and future. Gut Microbes.

[6] Evrensel A, Ceylan ME (2016). Fecal microbiota transplantation and its usage in neuropsychiatric disorders. Clin Psychopharmacol Neurosci.

[7] Cerovic M, Forloni G, Balducci C (2019). Neuroinflammation and the gut microbiota: possible alternative therapeutic targets to counteract Alzheimer’s disease?. Front Aging Neurosci.

[8] Ooijevaar RE, Terveer EM, Verspaget HW, Kuijper EJ, Keller JJ (2019). Clinical application and potential of fecal microbiota transplantation. Annu Rev Med.

[9] Castro I, Tasias M, Calabuig E, Salavert M (2019). Doctor, my patient has CDI and should continue to receive antibiotics. The (unresolved) risk of recurrent CDI. Rev Esp Quimioter.

[10] Isaac S, Scher JU, Djukovic A, Jiménez N, Littman DR, Abramson SB (2017). Short- and long-term effects of oral vancomycin on the human intestinal microbiota. J Antimicrob Chemother.

[11] Song JH, Kim YS (2019). Recurrent *Clostridium difficile* infection: risk factors, treatment, and prevention. Gut Liver.

[12] Deshpande A, Hurless K, Cadnum JL, Chesnel L, Gao L, Chan L (2016). Effect of fidaxomicin versus vancomycin on susceptibility to intestinal colonization with vancomycin-resistant *Enterococci* and *Klebsiella pneumoniae* in mice. Antimicrob Agents Chemother.

[13] Al-Nassir WN, Sethi AK, Li Y, Pultz MJ, Riggs MM, Donskey CJ (2008). Both oral metronidazole and oral vancomycin promote persistent overgrowth of vancomycin-resistant enterococci during treatment of *Clostridium difficile*-associated disease. Antimicrob Agents Chemother.

[14] Laffin M, Millan B, Madsen KL (2017). Fecal microbial transplantation as a therapeutic option in patients colonized with antibiotic resistant organisms. Gut Microbes.

[15] Woodworth MH, Hayden MK, Young VB, Kwon JH. The role of fecal microbiota transplantation in reducing intestinal colonization with antibiotic-resistant organisms: the current landscape and future directions. Open Forum Infect Dis. 2019;6. 10.1093/ofid/ofz288 PMC6667716 31363779

[16] Youngster I, Sauk J, Pindar C, Wilson RG, Kaplan JL, Smith MB (2014). Fecal microbiota transplant for relapsing *Clostridium difficile* infection using a frozen inoculum from unrelated donors: a randomized, open-label, controlled pilot study. Clin Infect Dis.

[17] Quraishi MN, Widlak M, Bhala N, Moore D, Price M, Sharma N (2017). Systematic review with meta-analysis: the efficacy of faecal microbiota transplantation for the treatment of recurrent and refractory *Clostridium difficile* infection. Aliment Pharmacol Ther.

[18] Iqbal U, Anwar H, Karim MA (2018). Safety and efficacy of encapsulated fecal microbiota transplantation for recurrent *Clostridium difficile* infection: a systematic review. Eur J Gastroenterol Hepatol.

[19] Hocquart M, Lagier J-C, Cassir N, Saidani N, Eldin C, Kerbaj J (2018). Early fecal microbiota transplantation improves survival in severe *Clostridium difficile* infections. Clin Infect Dis.

[20] Leung V, Vincent C, Edens TJ, Miller M, Manges AR (2018). Antimicrobial resistance gene acquisition and depletion following fecal microbiota transplantation for recurrent *Clostridium difficile* infection. Clin Infect Dis.

[21] DeFilipp Z, Bloom PP, Torres Soto M, Mansour MK, Sater MRA, Huntley MH (2019). Drug-resistant *E*. *coli* bacteremia transmitted by fecal microbiota transplant. N Engl J Med.

[22] Antibiotic resistance threats in the United States 2019. Centers for Diesase Control and Prevention; 2019. Available from: https://www.cdc.gov/drugresistance/biggest-threats.html.

[23] Johnston KJ, Thorpe KE, Jacob JT, Murphy DJ (2019). The incremental cost of infections associated with multidrug-resistant organisms in the inpatient hospital setting-a national estimate. Health Serv Res.

[24] Millan B, Park H, Hotte N, Mathieu O, Burguiere P, Tompkins TA (2016). Fecal microbial transplants reduce antibiotic-resistant genes in patients with recurrent *Clostridium difficile* infection. Clin Infect Dis.

[25] Singh R, de Groot PF, Geerlings SE, Hodiamont CJ, Belzer C, Berge IJMT (2018). Fecal microbiota transplantation against intestinal colonization by extended spectrum beta-lactamase producing *Enterobacteriaceae*: a proof of principle study. BMC Res Notes.

[26] Dubberke ER, Lee CH, Orenstein R, Khanna S, Hecht G, Gerding DN (2018). Results from a randomized, placebo-controlled clinical trial of a RBX2660-a microbiota-based drug for the prevention of recurrent *Clostridium difficile* infection. Clin Infect Dis.

[27] Orenstein R, Dubberke E, Hardi R, Ray A, Mullane K, Pardi DS (2016). Safety and durability of rbx2660 (microbiota suspension) for recurrent *Clostridium difficile* infection: results of the PUNCH CD study. Clin Infect Dis.

[28] Ray A, Jones C (2016). Does the donor matter? Donor vs patient effects in the outcome of a next-generation microbiota-based drug trial for recurrent *Clostridium difficile* infection. Future Microbiol.

[29] Blount KF, Shannon WD, Deych E, Jones C (2019). Restoration of bacterial microbiome composition and diversity among treatment responders in a phase 2 trial of RBX2660: an investigational microbiome restoration therapeutic. Open Forum Infect Dis.

[30] Mallick H, McIver L, Rahnavard A, Ma S, Zhang Y, Nguyen L, et al. Multivariable association in population-scale meta-omics studies. 10.1371/journal.pcbi.1009442 PMC8714082 34784344

[31] Segata N, Izard J, Waldron L, Gevers D, Miropolsky L, Garrett WS (2011). Metagenomic biomarker discovery and explanation. Genome Biol.

[32] Tankovic J, Leclercq R, Duval J (1993). Antimicrobial susceptibility of *Pediococcus* spp. and genetic basis of macrolide resistance in *Pediococcus acidilactici* HM3020. Antimicrob Agents Chemother.

[33] Mastro TD, Spika JS, Lozano P, Appel J, Facklam RR (1990). Vancomycin-resistant *Pediococcus acidilactici*: nine cases of bacteremia. J Infect Dis.

[34] Barton LL, Rider ED, Coen RW (2001). Bacteremic infection with *Pediococcus*: vancomycin-resistant opportunist. Pediatrics..

[35] Campedelli I, Mathur H, Salvetti E, Clarke S, Rea MC, Torriani S, et al. Genus-wide assessment of antibiotic resistance in *Lactobacillus* spp. Appl Environ Microbiol. 2019;85. 10.1128/AEM.01738-18 PMC6293106 30366997

[36] Ammor MS, Flórez AB, van Hoek AHAM, de Los Reyes-Gavilán CG, Aarts HJM, Margolles A (2008). Molecular characterization of intrinsic and acquired antibiotic resistance in lactic acid bacteria and bifidobacteria. J Mol Microbiol Biotechnol.

[37] Zarazaga M, Sáenz Y, Portillo A, Tenorio C, Ruiz-Larrea F, Del Campo R (1999). In vitro activities of ketolide HMR3647, macrolides, and other antibiotics against *Lactobacillus*, *Leuconostoc*, and *Pediococcus* isolates. Antimicrob Agents Chemother.

[38] Britt NS, Potter EM (2016). Clinical epidemiology of vancomycin-resistant *Enterococcus gallinarum* and *Enterococcus casseliflavus* bloodstream infections. J Glob Antimicrob Resist.

[39] Cai D, Sorokin V, Lutwick L, Liu W, Dalal S, Sandhu K (2012). *C*. *glycolicum* as the sole cause of bacteremia in a patient with acute cholecystitis. Ann Clin Lab Sci.

[40] Buu-Hoï A, Sapoetra A, Branger C, Acar JF (1982). Antimicrobial susceptibility of *Gemella haemolysans* isolated from patients with subacute endocarditis. Eur J Clin Microbiol.

[41] Lucas GM, Lechtzin N, Puryear DW, Yau LL, Flexner CW, Moore RD (1998). Vancomycin-resistant and vancomycin-susceptible enterococcal bacteremia: comparison of clinical features and outcomes. Clin Infect Dis.

[42] Tyrrell KL, Citron DM, Warren YA, Fernandez HT, Merriam CV, Goldstein EJC (2006). In vitro activities of daptomycin, vancomycin, and penicillin against *Clostridium difficile*, *C*. *perfringens*, *Finegoldia magna*, and *Propionibacterium acnes*. Antimicrob Agents Chemother.

[43] Chen EZ, Li H (2016). A two-part mixed-effects model for analyzing longitudinal microbiome compositional data. Bioinformatics..

[44] Jia B, Raphenya AR, Alcock B, Waglechner N, Guo P, Tsang KK (2017). CARD 2017: expansion and model-centric curation of the comprehensive antibiotic resistance database. Nucleic Acids Res.

[45] van der Maaten L, Hinton G (2008). Visualizing data using t-SNE. J Mach Learn Res.

[46] Gibson MK, Forsberg KJ, Dantas G (2015). Improved annotation of antibiotic resistance determinants reveals microbial resistomes cluster by ecology. ISME J.

[47] Pehrsson EC, Tsukayama P, Patel S, Mejía-Bautista M, Sosa-Soto G, Navarrete KM (2016). Interconnected microbiomes and resistomes in low-income human habitats. Nature..

[48] Aminov RI, Garrigues-Jeanjean N, Mackie RI (2001). Molecular ecology of tetracycline resistance: development and validation of primers for detection of tetracycline resistance genes encoding ribosomal protection proteins. Appl Environ Microbiol.

[49] Bryce A, Costelloe C, Hawcroft C, Wootton M, Hay AD (2016). Faecal carriage of antibiotic resistant Escherichia coli in asymptomatic children and associations with primary care antibiotic prescribing: a systematic review and meta-analysis. BMC Infect Dis.

[50] Lozupone CA, Stombaugh JI, Gordon JI, Jansson JK, Knight R (2012). Diversity, stability and resilience of the human gut microbiota. Nature..

[51] Vrieze A, Out C, Fuentes S, Jonker L, Reuling I, Kootte RS (2014). Impact of oral vancomycin on gut microbiota, bile acid metabolism, and insulin sensitivity. J Hepatol.

[52] Tomas ME, Mana TSC, Wilson BM, Nerandzic MM, Joussef-Piña S, Quiñones-Mateu ME, et al. Tapering courses of oral vancomycin induce persistent disruption of the microbiota that provide colonization resistance to *Clostridium difficile* and vancomycin-resistant *Enterococci* in mice. Antimicrob Agents Chemother. 2018;62. 10.1128/AAC.02237-17 PMC5923165 29530853

[53] Egland PG, Palmer RJ, Kolenbrander PE (2004). Interspecies communication in *Streptococcus gordonii*-*Veillonella atypica* biofilms: signaling in flow conditions requires juxtaposition. Proc Natl Acad Sci U S A.

[54] Johnson BP, Jensen BJ, Ransom EM, Heinemann KA, Vannatta KM, Egland KA (2009). Interspecies signaling between *Veillonella atypica* and *Streptococcus gordonii* requires the transcription factor CcpA. J Bacteriol.

[55] Scheiman J, Luber JM, Chavkin TA, MacDonald T, Tung A, Pham L-D (2019). Meta-omics analysis of elite athletes identifies a performance-enhancing microbe that functions via lactate metabolism. Nat Med.

[56] Ubeda C, Bucci V, Caballero S, Djukovic A, Toussaint NC, Equinda M (2013). Intestinal microbiota containing Barnesiella species cures vancomycin-resistant *Enterococcus faecium* colonization. Infect Immun.

[57] Human Microbiome Project Consortium (2012). Structure, function and diversity of the healthy human microbiome. Nature..

[58] Qin J, Li R, Raes J, Arumugam M, Burgdorf KS, Manichanh C (2010). A human gut microbial gene catalogue established by metagenomic sequencing. Nature..

[59] Ganji L, Alebouyeh M, Shirazi MH, Eshraghi SS, Mirshafiey A, Ebrahimi Daryani N (2016). Dysbiosis of fecal microbiota and high frequency of *Citrobacter*, *Klebsiella* spp., and *Actinomycetes* in patients with irritable bowel syndrome and gastroenteritis. Gastroenterol Hepatol Bed Bench.

[60] Saïdani N, Lagier J-C, Cassir N, Million M, Baron S, Dubourg G (2019). Faecal microbiota transplantation shortens the colonisation period and allows re-entry of patients carrying carbapenamase-producing bacteria into medical care facilities. Int J Antimicrob Agents.

[61] Tadesse DA, Zhao S, Tong E, Ayers S, Singh A, Bartholomew MJ (2012). Antimicrobial drug resistance in *Escherichia coli* from humans and food animals, United States, 1950-2002. Emerging Infect Dis.

[62] Bailey JK, Pinyon JL, Anantham S, Hall RM (2010). Commensal *Escherichia coli* of healthy humans: a reservoir for antibiotic-resistance determinants. J Med Microbiol.

[63] Lavigne J-P, Blanc-Potard A-B, Bourg G, Moreau J, Chanal C, Bouziges N (2006). Virulence genotype and nematode-killing properties of extra-intestinal *Escherichia coli* producing CTX-M beta-lactamases. Clin Microbiol Infect.

[64] McElvania TeKippe E, Burnham C-A.D. Evaluation of the Bruker Biotyper and VITEK MS MALDI-TOF MS systems for the identification of unusual and/or difficult-to-identify microorganisms isolated from clinical specimens. Eur J Clin Microbiol Infect Dis. 2014;33:2163–2171. 10.1007/s10096-014-2183-y 24962194

[65] Westblade LF, Garner OB, MacDonald K, Bradford C, Pincus DH, Mochon AB (2015). Assessment of reproducibility of matrix-assisted laser desorption ionization-time of flight mass spectrometry for bacterial and yeast identification. J Clin Microbiol.

[66] Clinical & Laboratory Standards Institute. M100 - performance standards for antimicrobial susceptibility testing: Clinical and Laboratory Standards Institute; 2019.

[67] Baym M, Kryazhimskiy S, Lieberman TD, Chung H, Desai MM, Kishony R (2015). Inexpensive multiplexed library preparation for megabase-sized genomes. PLoS One.

[68] Bolger AM, Lohse M, Usadel B (2014). Trimmomatic: a flexible trimmer for Illumina sequence data. Bioinformatics..

[69] Schmieder R, Edwards R (2011). Fast identification and removal of sequence contamination from genomic and metagenomic datasets. PLoS One.

[70] Bankevich A, Nurk S, Antipov D, Gurevich AA, Dvorkin M, Kulikov AS (2012). SPAdes: a new genome assembly algorithm and its applications to single-cell sequencing. J Comput Biol.

[71] Gurevich A, Saveliev V, Vyahhi N, Tesler G (2013). QUAST: quality assessment tool for genome assemblies. Bioinformatics..

[72] Seemann T (2014). Prokka: rapid prokaryotic genome annotation. Bioinformatics..

[73] Kurtz S, Phillippy A, Delcher AL, Smoot M, Shumway M, Antonescu C (2004). Versatile and open software for comparing large genomes. Genome Biol.

[74] Page AJ, Cummins CA, Hunt M, Wong VK, Reuter S, Holden MTG (2015). Roary: rapid large-scale prokaryote pan genome analysis. Bioinformatics..

[75] Price MN, Dehal PS, Arkin AP (2010). FastTree 2--approximately maximum-likelihood trees for large alignments. PLoS One.

[76] Letunic I, Bork P (2019). Interactive Tree Of Life (iTOL) v4: recent updates and new developments. Nucleic Acids Res.

[77] Segata N, Waldron L, Ballarini A, Narasimhan V, Jousson O, Huttenhower C (2012). Metagenomic microbial community profiling using unique clade-specific marker genes. Nat Methods.

[78] Oksanen J, Blanchet FG, Friendly M, Kindt R, Legendre P, McGlinn D, et al. vegan: community ecology package. 2019. Available from: https://CRAN.R-project.org/package=vegan.

[79] Paradis E, Claude J, Strimmer K (2004). APE: Analyses of phylogenetics and evolution in R language. Bioinformatics..

[80] Franzosa EA, McIver LJ, Rahnavard G, Thompson LR, Schirmer M, Weingart G (2018). Species-level functional profiling of metagenomes and metatranscriptomes. Nat Methods.

[81] Kaminski J, Gibson MK, Franzosa EA, Segata N, Dantas G, Huttenhower C (2015). High-specificity targeted functional profiling in microbial communities with ShortBRED. PLoS Comput Biol.

[82] McArthur AG, Waglechner N, Nizam F, Yan A, Azad MA, Baylay AJ (2013). The comprehensive antibiotic resistance database. Antimicrob Agents Chemother.

[83] Langmead B, Salzberg SL (2012). Fast gapped-read alignment with Bowtie 2. Nat Methods.

[84] Li H, Handsaker B, Wysoker A, Fennell T, Ruan J, Homer N (2009). The sequence alignment/map format and SAMtools. Bioinformatics..

