# Supplementary material for: Impact of investigational microbiota therapeutic RBX2660 on the gut microbiome and resistome revealed by a placebo-controlled clinical trial
Source: Microbiome. 2020 Aug 31;8:125. doi: 10.1186/s40168-020-00907-9 (PMC7457799; doi:10.1186/s40168-020-00907-9)
Supplement: Supplementary file 2 — Additional file 1: Figure S1. Taxonomic overview of patient stool samples at the genus level. Figure S2. Taxonomic shift by treatments (related Fig. 2). Figure S3. The effect of antibiotics prior to study drug on taxonomic shift by RBX2660 (related Fig. 2 and 3). Figure S4. Bray-Curtis dissimilarities between patients and respective RBX2660 (DR) or other random RBX2660 (DO). Figure S5. Changes in the Bray-Curtis dissimilarities between a patient and corresponding donor. Figure S6. Transplantation indices (TIs) and pseudo transplantation indices (pTIs). Figure S7. Additional discriminative features of the obstinate patients (related Fig. 3). Figure S8. Comparison of resistome compositions. Figure S9. Random forest classifier successfully distinguished between donor and patient baseline resistomes (related Fig. 5). Figure S10. Average nucleotide identity (ANI) and core genome phylogeny of E. coli isolates. Figure S11. Average nucleotide identity (ANI) and core genome phylogeny of VRE isolates. Figure S12. Antibiotic susceptibility testing (AST). [file 40168_2020_907_MOESM1_ESM.docx]

**Figure S1. Taxonomic overview of patient stool samples at the genus level.** Genus composition of RBX2660 products was added to the corresponding recipient. **a** Patients who received 2 doses of placebo. **b** Patients who received 1 dose RBX2660 and 1 dose of placebo. **c** Patients who received 2 doses of RBX2660. Patients R0-01, R0-20, and R2-09, who had only the baseline specimen (due to early rCDI) that exhibited insufficient sequencing depth for the analysis of taxonomic structure after decontamination of human reads, were omitted. Patient R2-17 was also omitted from this analysis due to incomplete donor RBX2660 information.

**Figure S2. Taxonomic shift by treatments (related Fig. 2). a** Distribution of inter-subjects Bray-Curtis dissimilarities (yellow) and intra-subject dissimilarities (green) regarding taxonomic structures by treatment groups (column) and by time frames (rows). The similar distribution of inter-subjects and intra-subject dissimilarities during the first week in all conditions suggests significant taxonomic shifts in early stage regardless of the dose of RBX2660. Wilcoxon rank sum tests were performed to compare inter-subjects and intra-subject dissimilarities of each treatment group during each time frame. **b** Comparison of intra-subject dissimilarities of placebo (grey), single (red), and double RBX2660 recipients (blue) during each time frame with Kruskal-Wallis rank sum tests.

**Figure S3. The effect of antibiotics prior to study drug on taxonomic shift by RBX2660 (related Fig. 2 and 3). a** RBX2660 products exhibited significantly higher alpha diversity than patient baseline samples (Wilcoxon signed-rank test). Changes in alpha diversity (**b**) and Bray-Curtis dissimilarity (**c**) to corresponding RBX2660 of vancomycin recipients. Changes in the diversity and dissimilarity were still statistically significant for the first week after study drug (black, Wilcoxon signed-rank test) without the metronidazole and fidaxomicin recipients, and RBX2660 recipients showed a more dynamic decrease in Bray-Curtis dissimilarity than placebo recipients after the first week (red, Kruskal-Wallis test). **d** Transplantation index of patients on day 7 and 60. Horizontal dash lines indicate the threshold of taxonomic transplantation (Fig. 2a). Violet, vancomycin; yellow, metronidazole; green, fidaxomicin. *P ≤ 0.05, **P ≤ 0.01, ***P ≤ 0.001, ****P ≤ 0.0001.

**Figure S4. Bray-Curtis dissimilarities between patients and respective RBX2660 (D_R_) or other random RBX2660 (D_O_).** Pairwise comparisons of all D_R_s and D_O_s of placebo (gray), single dose (red), and double dose recipients (blue) in each time point were simultaneously performed (Wilcoxon signed-rank test with Benjamini-Hochberg FDR correction, FDR < 0.05). Dissimilarities of double dose recipients include both dissimilarities to the first and second RBX2660 doses. *P ≤ 0.05, **P ≤ 0.01, ***P ≤ 0.001, ****P ≤ 0.0001.

**Figure S5.** **Changes in the Bray-Curtis dissimilarities between a patient and corresponding donor** after **a** single RBX2660 and **b-c** double RBX2660 treatments. Changes in taxonomic structures of gut microbiota were significant for the first week after treatments (Kruskal-Wallis test, ***P < 0.001). There were no statistically significant differences between patients who experienced recurrent *Clostridioides difficile* infection (rCDI, white) and other successful patients (gray) at all time points (Wilcoxon signed-rank test with Benjamini-Hochberg FDR correction, FDR < 0.05). D1, the first dose; D2, the second dose. **d** Relative abundance of *Klebsiella pneumoniae* in all patients. Patients who experienced rCDI (white) exhibited significantly higher *K. pneumoniae* abundance than treatment-success patients (gray). **e** Relative abundance comparison of *K. pneumoniae* between treatment-failture and -success patients in placebo recipients. Relative abundance comparison of **d** *Akkermansia muciniphila* and **e** *Leptotrichia wadei* that were identified by MaAsLin2 as features associated with treatment-failures of single RBX2660 dose recipients. MaAsLin2 could not identify any taxonomic feature associated with treatment outcome from double RBX2660 recipients.

**Figure S6. Transplantation indices (TIs) and pseudo transplantation indices (pTIs).** Pairwise comparisons of all TIs and pTIs of placebo (gray), single dose (red), and double dose recipients (blue) in each time frame were simultaneously performed (Wilcoxon signed-rank test with Benjamini-Hochberg FDR correction, FDR < 0.05). Indices of double dose recipients include both indices for the first and second RBX2660 doses. *P ≤ 0.05, **P ≤ 0.01, ***P ≤ 0.001, ****P ≤ 0.0001.

**Figure S7. Additional discriminative features of the obstinate patients (related Fig. 3). a** Comparison of transplantation indices of double RBX2660 recipients for the first and the second RBX2660 at day 7 and day 60. R2-21 exhibited lower engraftment index for the first dose but higher engraftment index for the second dose of RBX2660 than placebo patients at day 60 (white square). **b** Metabolic pathway features of the double RBX2660 recipients whose taxonomic structures were engrafted and maintained until day 60 (green) and other non-engrafted patients (purple). Yeast-specific metabolic pathways were marked in red. GLCMANNANAUT-PWY, superpathway of N-acetylglucosamine, N-acetylmannosamine and N-acetylneuraminate degradation; GALACT-GLUCUROCAT-PWY, superpathway of hexuronide and hexuronate degradation. At baseline, **c** Shannon index (Wilcoxon signed-rank test, P = 0.41), **d** *Proteobacteria* (P = 0.79), **e** *Bacteroidetes* (P = 0.92), **f** *Firmicutes* (P = 0.32), and **g** the ratio between *Bacteroidetes* and *Firmicutes* (P = 0.92) were not significantly different between the engrafted and non-engrafted double RBX2660 recipients.

** Figure S8. Comparison of resistome compositions. a** Alpha diversity of baseline patient resistomes was comparable to that of RBX2660 (only patients who received vancomycin, P = 0.066; all patients, P = 0.180). **b** Baseline patient resistomes had a greater antibiotic resistant gene (ARG) reads per kilobase per million sample reads (RPKM, Wilcoxon signed-rank test). ****P ≤ 0.0001. **c** Principal coordinates analysis (PCoA) of resistome composition showed a clustering of RBX2660 (white). Baseline resistomes of metronidazole and fidaxomicin recipients were more closely clustered with other baseline resistomes of vancomycin recipients (P = 0.0120, PERMANOVA and pairwise comparison with Pillai-Bartlett non-parametric trace and Benjamini-Hochberg FDR correction) than RBX2660 (P = 0.0015). **d** Individual loads of an antibiotic resistant gene (ARG) in a treatment arm were averaged. ARGs whose average portion in the treatment arm was smaller than 2% were combined as “Rare ARGs.”

**
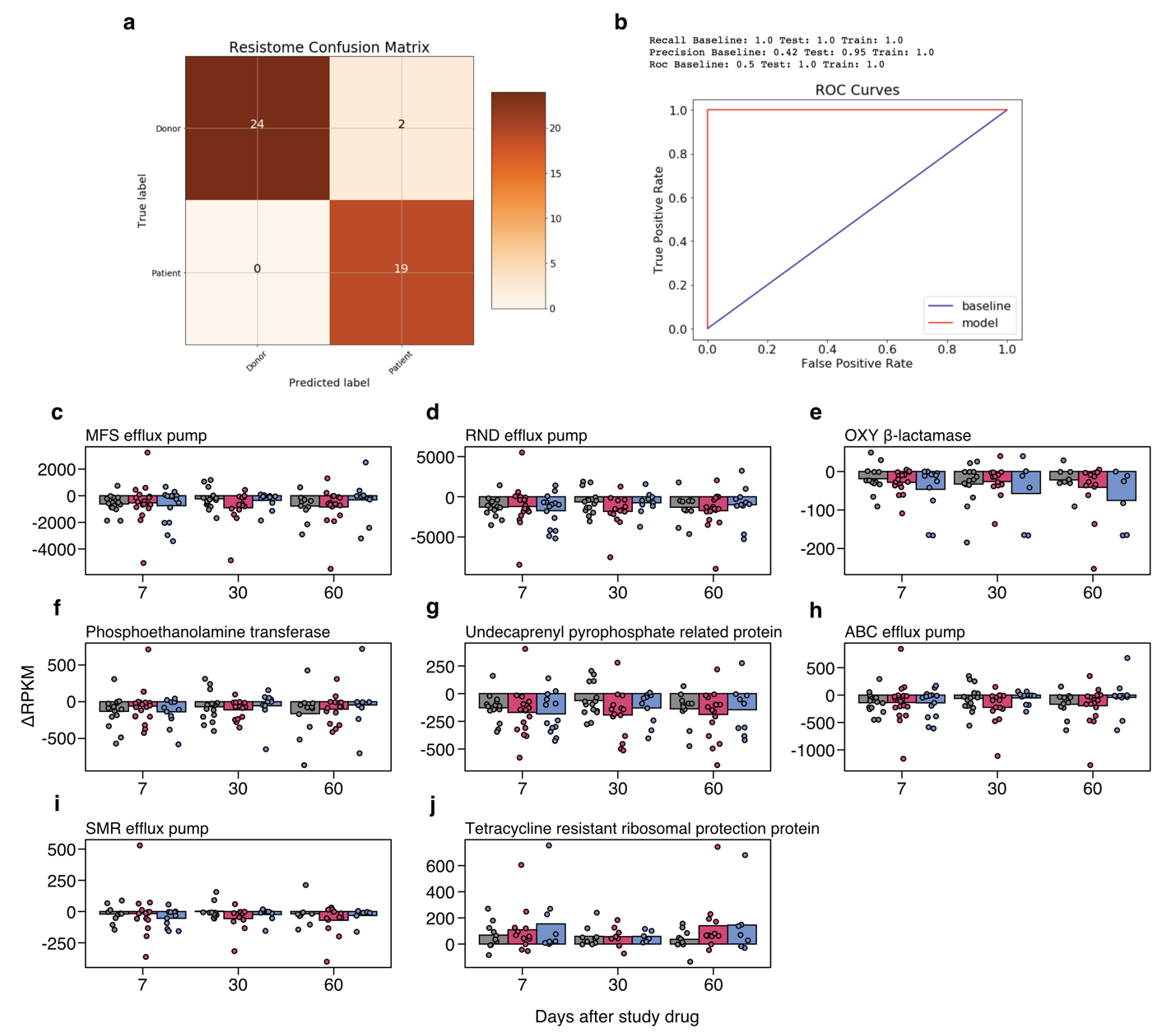
**

**Figure S9. Random forest classifier successfully distinguished between donor and patient baseline resistomes (related Fig. 5).** **a** Resistome confusion matrix depicting predicted and true labels for the test set (n = 45). All but 2 samples were correctly categorized. **b** A receiver operating characteristic (ROC) curve showed high recall, precision, and area under curve (AUC) for the model. **c–j** Individual changes in abundance (reads per kilobase per million sample reads, RPKM) of selected antibiotic resistant genes from baseline were similar among patients in the three treatment groups. Individual changes of the two RBX2660-origin beta-lactamases, whose baseline abundances were near-zero, were described in Fig. 5i and 5j.

**Figure S10. Average nucleotide identity (ANI) and core genome phylogeny of *E. coli* isolates. a** ANI for all *E. coli* isolates pairwise comparisons. All isolates show at least 97% pairwise identity. **b** Core genome phylogeny of *E. coli* isolates with 24 NCBI reference strains and *E. fergusonii* as outgroup. Right panel indicates *E. coli* phylogroup. Isolates originated from the same patient or donor were labelled in the same color. Reference strains were marked in black and bold. Small colored squares indicate isolates from donor product that was administered to multiple patients, where the color of the text and squares correspond to the different patients.

**Figure S11.** **Average nucleotide identity (ANI) and core genome phylogeny of VRE isolates. a** ANI for all VRE isolates pairwise comparisons. All isolates show at least 99.43% pairwise identity. **b** Core genome phylogeny of VRE isolates, with 4 NCBI reference VRE strains and V583 *Enterococcus faecalis* as outgroup. Isolates originated from the same patient were labelled in the same color. Reference strain names were marked in black.

**Figure S12. Antibiotic susceptibility testing (AST). a** *E. coli* and **b** VRE isolates. AST showed whether isolates are susceptible (white) or intermediate/resistant (black) to a variety of antibiotics. Source of isolate and specific patient of origin were depicted in the sidebars. RBX2660-derived antibiotic resistant organisms engrafted in patients were colored as their corresponding patient. RBX2660 samples without a corresponding patient are otherwise denoted as “RBX2660.”
